# Effect of proximal translation of the osteotomized tibial tuberosity during tibial tuberosity advancement on patellar position and patellar ligament angle

**DOI:** 10.1186/s12917-017-0942-6

**Published:** 2017-01-09

**Authors:** Jack D. Neville-Towle, Mariano Makara, Kenneth A. Johnson, Katja Voss

**Affiliations:** University Veterinary Teaching Hospital, Sydney, Faculty of Veterinary Science, University of Sydney, Sydney, NSW 2006 Australia

**Keywords:** Cranial cruciate ligament, Tibial tuberosity advancement, Patellar position, Canine

## Abstract

**Background:**

Cranial cruciate ligament insufficiency is a common orthopaedic problem in canine patients. This cadaveric and radiographic study was performed with the aim of determining the effect of proximal translation of the tibial tuberosity during tibial tuberosity advancement (TTA) on patellar position (PP) and patellar ligament angle (PLA).

**Results:**

Disarticulated left hind limb specimens harvested from medium to large breed canine cadavers (*n* = 6) were used for this study. Limbs were mounted to Plexiglass sheets with the stifle joint fixed in 135° of extension. The quadriceps mechanism was mimicked using an elastic band. Medio-lateral radiographs were obtained pre-osteotomy, after performing TTA without proximal translation of the tibial tuberosity, and after proximal translation of the tibial tuberosity by 3mm and 6mm. Radiographs were blinded to the observer for distance of tibial tuberosity proximalization following radiograph acquisition. Three independent observers recorded PP and PLA (tibial plateau method and common tangent method). Comparisons were made between the stages of proximalization using repeated measures ANOVA. Patellar position was found to be significantly more distal than pre-osteotomy, if the tibial tuberosity was not translated proximally (*P* = 0.001) and if it was translated proximally by 3mm (*P* = 0.005). The difference between pre-osteotomy PP and 6mm proximalization was not significant. The PLA was significantly larger if the tibial tuberosity was not translated proximally compared to tibial tuberosity proximalization of 6mm using the tibial plateau and the common tangent methods (*P* = 0.006 and *P* = 0.015 respectively).

**Conclusions:**

Proximalizing the tibial tuberosity during TTA helps in maintaining vertical position of the patella in the patellar groove. Proximalization of the tibial tuberosity reduces PLA when compared to TTA without tibial tuberosity proximalization.

## Background

Cranial cruciate ligament disease is the most commonly diagnosed cause of osteoarthritis and lameness in the canine stifle [1–4]. Given the prevalence of disease, development of treatment techniques, including various tibial osteotomies have been described over the past 25 years [5–9]. These procedures are intended to change stifle dynamic joint biomechanics rather than directly repair the cranial cruciate ligament and include the cranial tibial wedge osteotomy [6] the tibial plateau levelling osteotomy [7], the tibial tuberosity advancement [8], and the triple tibial osteotomy [9].

The TTA technique was first described in 2002 and aims to neutralise cranial shear force of the tibia by reducing the patellar ligament angle to 90° [8]. Reduction of the patellar ligament angle is achieved by osteotomizing and advancing the tibial tuberosity cranially. Clinically, cranial advancement of the tibial tuberosity can be performed either with or without concurrent proximal translation of the distal end of the tibial tuberosity. With the original TTA technique, the advanced tibial tuberosity is stabilized using a cage, which prevents collapse of the osteotomy, and a tension band plate, which counteracts the tensile forces exerted on the tibial tuberosity by the quadriceps muscles and the patellar ligament [8, 10].

In recent years, several modifications of the TTA technique have been described. One of these modifications is the modified Maquet technique or more recently the modified Maquet procedure (MMP) [5, 11–13]. The original Maquet technique was described for humans in 1976 and involved advancement of tibial tuberosity with the aim of reducing retropatellar pressure in patients with femoropatellar osteoarthritis [13]. Subsequently, the concept of the MMP involved a proximal tibial osteotomy similar to that described by Montavon et al. [8] but instead of using a plate to counteract the tensile forces exerted on the tibial tuberosity by the quadriceps mechanism, these forces are counteracted by performing an incomplete osteotomy that leaves a cortical hinge distally [5]. Collapse of the osteotomy can be prevented by insertion of a TTA cage or another spacer and a number of variations of this procedure have been reported [12, 14, 15]. Initial clinical and biomechanical studies suggest that the technique is biomechanically sound and may be safely applied in clinical patients [5, 11, 16].

With the original TTA technique, several millimetres of proximal tibial tuberosity translation has been recommended to maintain vertical position of the patella during advancement [10]. The TTA technique can also be used to correct pre-existing patella baja or alta by shifting the tibial tuberosity proximally or distally, respectively [17]. With the MMP, it is not possible to shift the tibial tuberosity proximally as the technique relies on the presence of a distal cortical hinge around which the tibial tuberosity is rotated cranially. Rotation of the tibial tuberosity around the distal cortical hinge could result in patella baja as the insertion of the patellar ligament moves craniodistal to the patella during advancement. Studies reported in the human literature have shown that there is a 15% decrease in patella height after a similar procedure with 26% of patients meeting new-onset patella baja criteria postoperatively [18, 19].

A recent trigonometric study has shown that there is a discrepancy between preoperative PLA measurements and the actual PLA achieved by advancing the tibial tuberosity as calculated [20]. The findings of this study have since been substantiated clinically with actual tibial tuberosity advancement achieved during the MMP being 30% lower than the calculated wedge size used [14]. This discrepancy is worse in dogs with a steep tibial plateau and when larger cage sizes are used. The consequence of this is a potential failure to achieve a 90^0^ PLA postoperatively [20]. Discrepancies have also been noted between the tibial plateau slope and common tangent methods of determining PLA at varying degrees of stifle extension [21]. This highlights the importance of consistent limb positioning in assuring the accuracy of these measurements [21–25]. The effect of proximalization of the tibial tuberosity during advancement on postoperative PLA is currently unknown.

The aims of this study were to provide an ex vivo comparison of PP and PLA following TTA with varying degrees of proximal translation of the tibial tuberosity (0mm, 3mm and 6mm). We hypothesized that proximalization of the tibial tuberosity during TTA would have a significant effect on PP and the PLA.

## Results

The breeds of the dogs used for this study were mixed-breed (*n* = 3), Labrador (*n* = 1), Rottweiler (*n* = 1) and Australian Cattle Dog (*n* = 1). Body weight ranged from 23.2 to 41.0kg with a mean of 33.0 (±6.4 kg). Cage sizes used based on pre-osteotomy measurements were 12mm (*n* = 3), 10.5mm (*n* = 2) and 9mm (*n* = 1). Radiographic assessment of the 3mm and 6mm tibial tuberosity proximalizations showed that the 3mm stage had a mean proximalization of 3.17 (± 0.55mm) and the 6mm stage a mean of 5.90 (± 0.46mm) respectively.

### Patellar position measurements

Inter-observer agreement for the PP measurements was 0.9 (95% CI: 0.723–0.960).

**Fig. 1:**
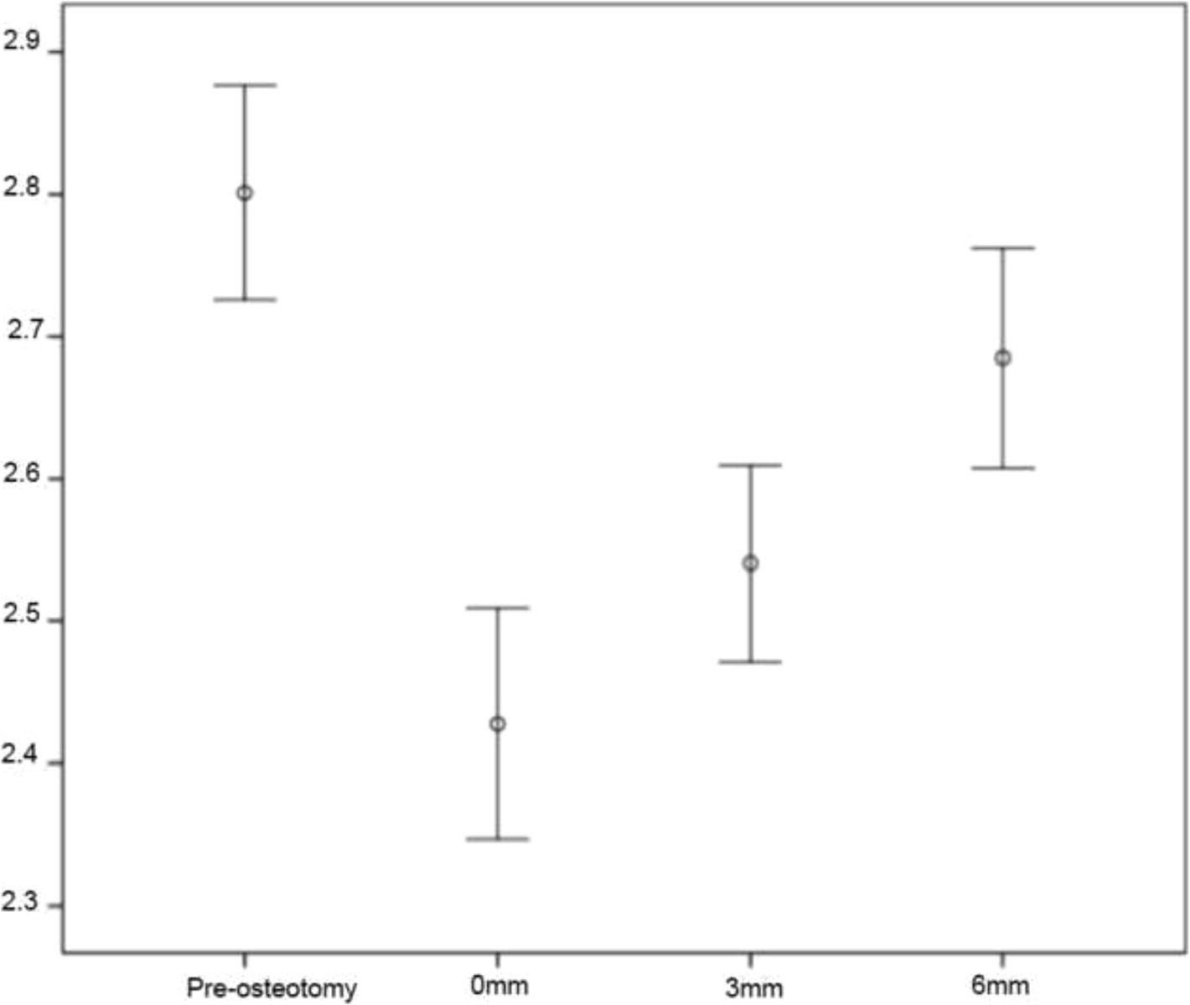
Patellar Position Error chart (+/ 1 SE) representing the change in patella position (mm) between tibial tuberosity proximal translation stages. Statistically significant difference was observed between the pre-osteotomy and 0mm, pre-osteotomy and 3mm, 0mm and 6mm and 3mm and 6mm stages

Mean pre-osteotomy PP measurement was 28.0mm (± 0.32). This distance was reduced post-osteotomy to 24.3mm (±0.34) if the tibial tuberosity was not proximally translated, 25.4mm (±0.29) if it was proximally translated by 3mm, and 26.8mm (±0.33) if it was proximally translated by 6mm (Fig. 1). Bonferroni pair-wise comparisons indicated that the patella was positioned significantly lower than pre-osteotomy measurements if the tibial tuberosity was not translated proximally (*P* = 0.001) and if it was translated proximally by 3mm (*P* = 0.005). Difference between 3mm and 6mm proximal translations was also significant (*P* = 0.002) whilst the difference between pre- and postoperative patellar position with the tibial tuberosity proximalized by 6mm was not. The difference between 0mm and 3mm proximal translations was also not significant.

### Patellar ligament angle measurements (PLAtangent and PLAslope)

There was excellent interobserver agreement for both PLA measurements with an agreement of 0.922 (95% CI: 0.822–0.966) for PLAslope and 0.925 (95% CI: 0.66–0.975) for PLAtangent.

**Fig. 2:**
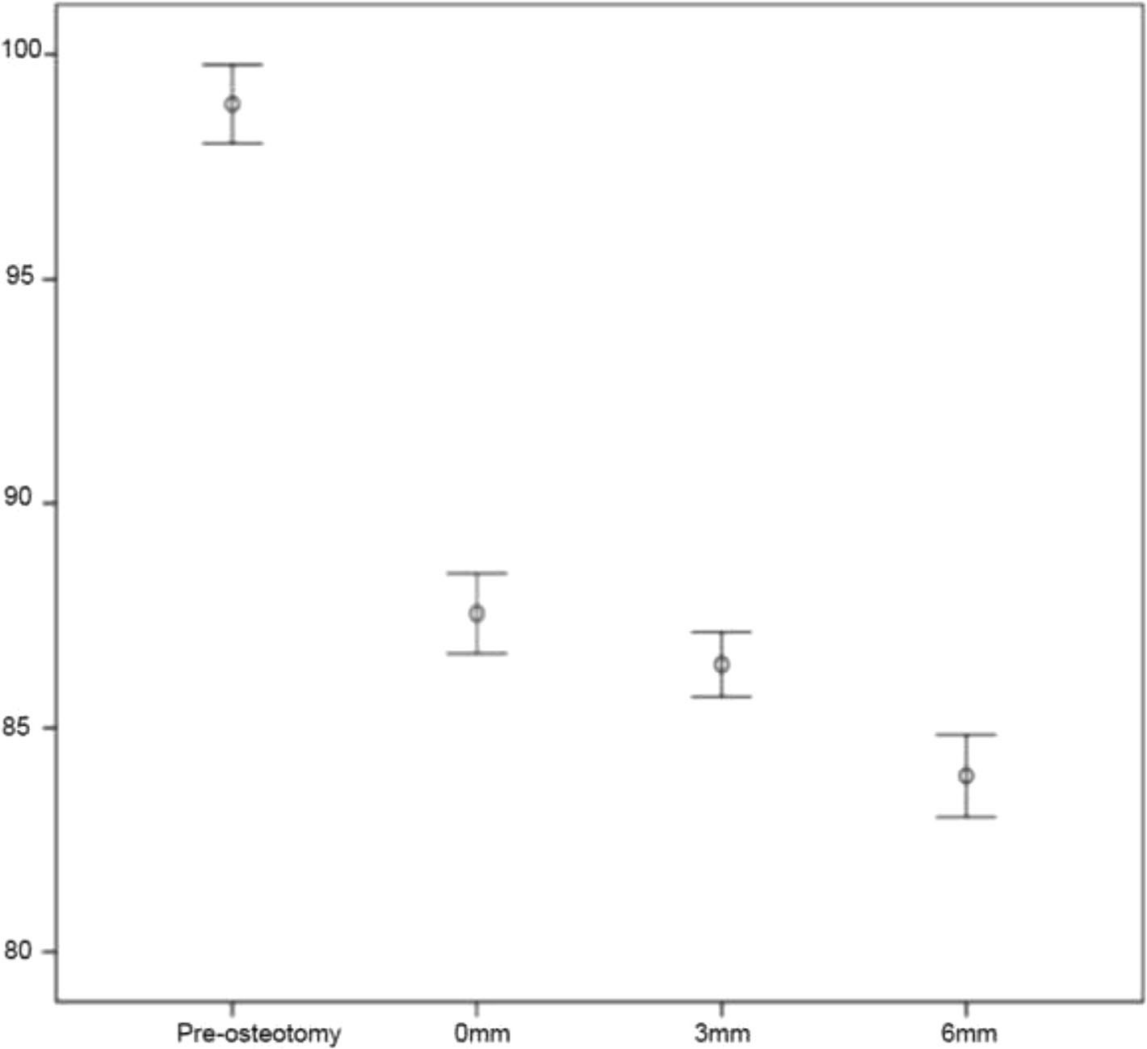
Patellar Ligament Angle, as measured by the common tangent technique. Error chart (+/- 1 SE) representing the change in PLAtangent (^o^) between tibial tuberosity proximal translation stages. Statistically significant difference was observed between the 0mm and 6mm stage and 3mm and 6mm stage

**Fig. 3:**
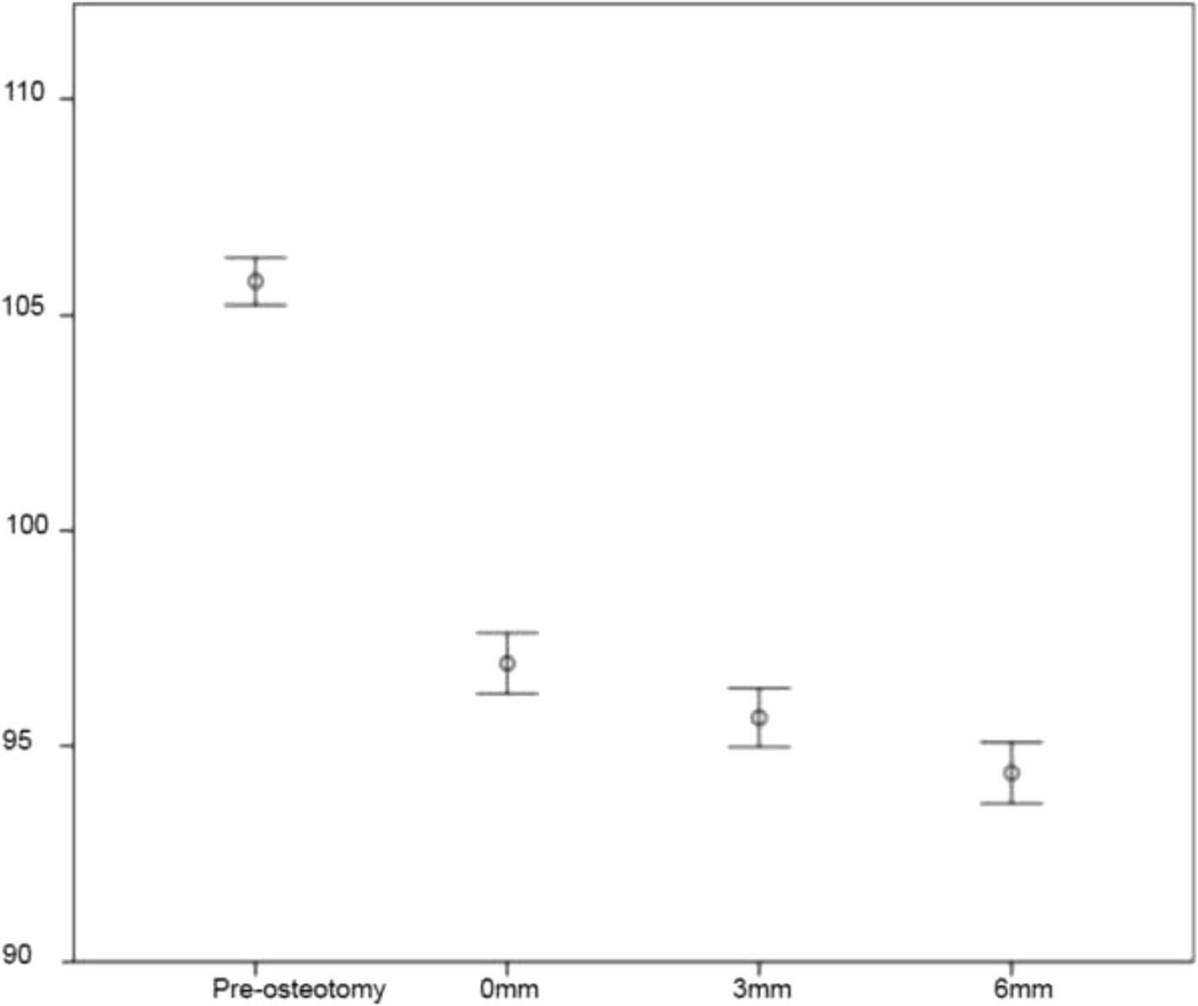
Patellar Ligament Angle, as measured by the tibial plateau slope technique. Error chart (+/- 1 SE) representing the change in PLAslope (^o^) between tibial tuberosity proximal translation stages. Statistically significant difference was observed between the 0mm and 6mm stage

Both PLAtangent and PLAslope decreased with increased proximal translation of the tibial tuberosity. Mean preoperative PLAtangent was 98.97° (± 3.41). Mean postoperative PLAtangent was 87.46° (±3.47), 86.42° (±2.88) and 83.85° (±3.67) at 0mm, 3mm and 6mm of proximal translation of the tibial tuberosity, respectively (Fig. 2). Mean preoperative PLAslope was 105.78° (±2.58). Postoperative PLAslopes were 96.92° (±3.40), 95.65° (±3.26) and 94.37° (±3.39) at 0mm, 3mm and 6mm of proximal translation (Fig. 3). Differences between groups were significant (*P* = 0.001 for PLAtangent; *P* < 0.001 for PLAslope). Pair-wise comparisons using Bonferroni adjustment revealed significant differences between PLAslope (*P* = 0.006) and PLA tangent angles (*P* = 0.015) at 0mm and 6mm of tibial tuberosity proximalization. The differences between 3mm and 6mm were significantly different for PLAtangent (*P* = 0.002), but not PLAslope. The difference between the 0mm and 3mm stages was not significant for PLAslope or PLAtangent.

## Discussion

This study has shown that failure to translate the tibial tuberosity proximally during TTA results in significant distal displacement of the patella as compared to shifting the tibial tuberosity proximally by 6mm. Additionally, post-osteotomy PLAs (PLAtangent and PLAslope) were larger when the tibial tuberosity was not proximally translated.

Several methods exist to quantify PP. We chose the Blackburne and Peel [26] method because it has been shown to have less interobserver error when compared to other commonly used techniques [27]. With the specimen in a fixed position between radiographs the common tangent of the femoro-tibial articulation was considered a consistent reference from which to measure [21, 28, 29]. We found excellent interobserver agreement using this method.

Significant distal patella displacement occurred when the tibial tuberosity was not proximalized or only proximalized by 3mm, compared to pre-osteotomy measurements. This supports the description to proximalize the tibial tuberosity during TTA in order to maintain patellar position [10]. In the group of dogs in this study with a mean weight of 33kg, the mean distal displacement occurring without translation of the tibial tuberosity as compared to pre-osteotomy was 3.7mm. The clinical implication of distal patella displacement as shown in this study is unknown. Additionally, a relationship between patella baja and increased incidence of congenital lateral patellar luxation has been described in dogs [30]. From our data, it is not possible to state whether the observed degree of distal patellar displacement could increase the risk for lateral patellar luxation postoperatively. The degree of distal patellar displacement during TTA is likely to be larger when larger cage sizes are used because the patellar ligament insertion will be pulled increasingly more distally with increasing degrees of tibial tuberosity advancement.

The exact distance of proximal translation necessary to maintain patellar position in clinical cases is unknown and does depend on size of the animal, length of the osteotomy, shape of the proximal tibia, and cage size used. In this study, if the tibial tuberosity was proximalized by 6mmthe patella was still displaced distally as compared to pre-osteotomy, although this difference was no longer statistically significant. Patella alta was therefore not a result in any of our specimens. The 3mm and 6mm tibial tuberosity proximalization distances were selected so as to mimic the author’s (KV) clinical experience with the described TTA technique [8, 10].

Clinically, it seems that in some cases there is a discrepancy between preoperative measurements and postoperative outcome in that the postoperative PLAs tend to be larger than expected, resulting in inadequate advancement of the tibial tuberosity and insufficient dynamic stability of the stifle joint. One study based on a MMP type procedure found that with larger cage sizes and steeper tibial plateau slopes, a larger discrepancy between preoperative measurements and postoperative outcome can be expected [20]. This seems to occur because the direction of the preoperative measurements along the axis of the tibial plateau or common tangent differs to the direction of advancement achieved clinically when the tibial tuberosity is rotated around its distal attachment. Clinically, the direction of advancement is more distal than the direction calculated on preoperative measurements, creating a discrepancy in the distance required to sufficiently advance the tibial tuberosity to the preoperative plan. When proximalizing the tibial tuberosity during the advancement, the tibial tuberosity also shifts more cranially which somewhat offsets the loss of advancement. This effect was significant in the current study between the 0mm and the 6mm proximalization stages. The actual change in PLA between the 0mm and 6mm proximalizations was approximately 2.5° for the PLAslope and 4° for the PLAtangent in the cadavers evaluated in this study. Based on the current literature, it is unknown whether a relatively small increase in PLA would be of clinical importance, as good clinical outcomes have been reported even with suboptimal post-operative PLA [31].

While no consistent or significant relationship between PLA at 0mm and 3mm proximalizations was found, there was a continuous decrease in PLA as the tibial tuberosity was proximalized. The decrease in PLA with increasing proximalization was not linear. There was a smaller decrease in PLA between the 0mm and 3mm stage than between the 3mm and 6mm stage. This was most likely due to the cage shifting proximal to a larger degree from the 0mm to the 3mm stage of proximalization than from the 3mm to 6mm stage in most specimens. Even slight proximal displacement of the cage within the osteotomy wedge will naturally result in reduced advancement of the tibial tuberosity. Whilst we acknowledge that the observed movement of the cages between conditions is a limitation of the accuracy of the study, it should not have influenced the significance of our results; proximal displacement of the cage results in reduction of the advancement, which would have lessened rather than increased the effect of proximalization of the tuberosity. Tilting of the cage ears and subsequent proximal dislodgement of the cage could likely have been avoided by tilting the cage ears prior to fixing the caudal cage ear to the tibial metaphysis.

The same pattern was observed for the PLAslope and PLAtangent methods. There is currently no definitive study in the veterinary literature to determine whether we should aim for a 90° PLAtangent or PLAslope value when determining TTA cage size preoperatively. It has been suggested that the common tangent method represents a more functionally accurate measurement than the tibial slope method [32] however conversely, the common tangent method has also been found to represent a lesser degree of advancement [33]. In this study, using a compromise of cage size between the two methods, it was expected that there was an overall apparent overcorrection when using post-osteotomy common tangent measurements, and an undercorrection when using the tibial plateau slope measurements. The difference between each of the measurements was as high as 11° within one specimen in this study. A recent study demonstrated that 70% of dogs that had undergone a TTA and had a mean postoperative PLAtangent of 89° continue to have cranial tibial subluxation when weight-bearing, suggesting that further in vivo studies need to be performed to determine which of the measurements would result in optimal clinical outcomes [34]. Certainly, our results suggest that if an MMP type procedure is performed using the same pre-operative planning techniques [33] to those recommended for the original TTA procedure [8, 10], this may result in under-advancement of the tibial tuberosity.

There were several limitations of the study that must be acknowledged. Although the blinding method used did not allow assessment of the degree of proximalization of the tibial tuberosity itself, it became apparent during the study that in the proximalized stages the cage ear insert of the cage tended to tilt, and to some observers it was apparent that these radiographs represented specimens in which there was a proximalization of the tibial tuberosity. It was not apparent though whether the proximalization was 3mm or 6mm as the tilting of the cage ear occurred between completing the osteotomy and proximalization of the tibial tuberosity. Another limitation is that the tension of the rubber band tensioning device in the patellar ligament was not standardised or measured, however there was a narrow distance range (34–43mm) between the end of the suture loops and pin with a standard four passes between these anchoring points, so that the tension exerted by the rubber band would have been similar between specimens. The procedures and radiographs were carried out in one session per specimen and it is unlikely that stretching of the rubber band would have resulted in decreasing tension during the experiment. Whilst the small sample size of this study is a potential limitation, the excellent interobserver agreement between measurements in all groups as well as statistically significant differences demonstrated between different tibial tuberosity positions show that our results can be interpreted as an indication of the clinical result of the TTA on patellar position and patellar ligament angle. The small sample size of six limbs was selected given the close standardization of the methods and similar conformation of the limbs, minimizing confounding variables.

In our study, variables that could affect the degree of advancement of the tibial tuberosity with a given cage size included the length of the osteotomy, tibia plateau slope, shape of the tibial tuberosity and position of the cage in relation to the level of tibial tuberosity. Whilst we were not able to control these variables in our study, similarly to the clinical situation, our statistical analysis meant that each specimen was compared to itself and therefore interference of these variables with the results was avoided. Patellar position measurements may have also been affected by a number of factors in our study. Positioning the limbs without mimicking weight-bearing, as well as the removal of surrounding soft tissues may have changed the patella position compared to an in vivo scenario however the standardization of this methodology made the results comparable.

The results reported in this study have been obtained with large breed specimens and as such, the magnitudes of patella position and patella ligament angle measurements may not be directly comparable in smaller breed dogs or dogs with different tibial conformations. Similarly, the use of solely left pelvic limbs in our study meant that comparison between right and left was not possible, and therefore symmetry in our results could not be assessed.

## Conclusions

In this report, we show that proximalization of the osteotomized tibial tuberosity during the TTA procedure in a cadaveric model is necessary to maintain vertical patellar position. Proximalization of the tibial tuberosity also reduces post-osteotomy PLA as compared to no proximalization and is another factor that should be considered during preoperative planning.

## Methods

### Cadaver preparation

Left hind limbs of six large-breed dogs were included in the study. The dogs had been euthanized for reasons unrelated to the study and subsequently stored at –20 °C. Use of the cadavers was in accordance with the guidelines of the animal ethics committee of the institution. The left hind limbs were disarticulated at the coxofemoral joint once the cadavers were thawed at room temperature. The limbs were prepared by removing skin between the tarsal and coxofemoral joints together with the biceps femoris, hamstrings, and gastrocnemius muscles. The quadriceps muscle was transected approximately 2cm proximal to the insertion on the patella and removed proximally. The joint capsule, collateral ligaments, patella and patellar ligament were left intact.

**Fig. 4:**
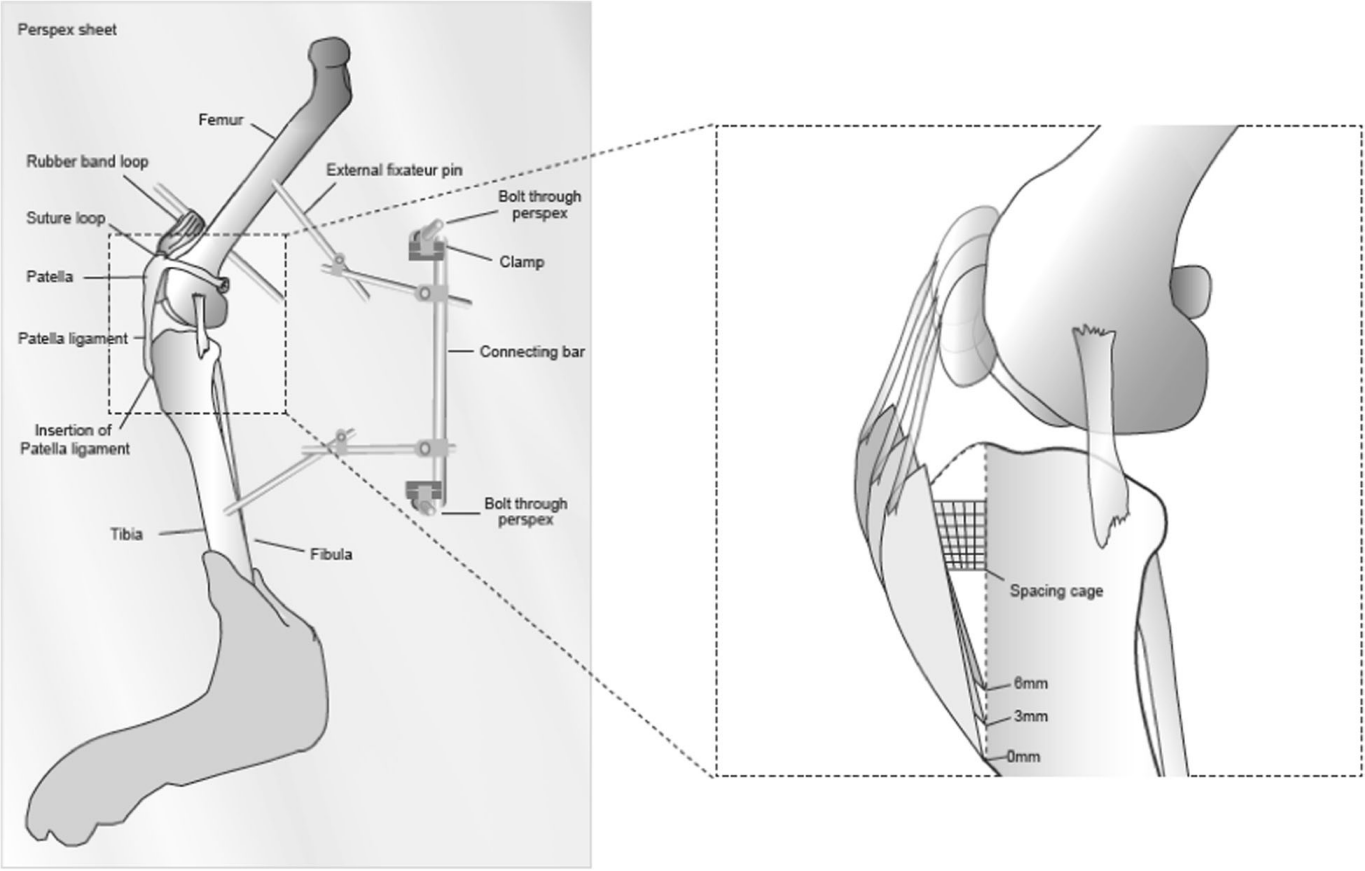
Representation of apparatus and specimen positioning. Illustration representing the positioning and apparatus used to prepare each specimen (*left*) and the positioning of the tibial tuberosity during each of the proximal translation stages of the study (*right*). The stifle specimens were mounted to the Plexiglass base in 135° of extension

Two 3.2mm end-threaded half pins were placed centrally in the diaphysis of the tibia and the femur in a caudocranial direction to create anchor points for mounting the limb to a polymethylmethacrylate (Plexiglass) base caudally. A third pin was placed into the distal third of the femur, also in a caudocranial direction; the pin protruded cranially to create an anchor point for the patella proximally. The limbs were mounted to the Plexiglass sheet using additional pins and small external fixator clamps^1^ with the medial side of the limb facing away from the sheet (Fig. 4). Stifle joint angle was measured with a goniometer, using the following landmarks; greater trochanter of the femur, midpoint between the fibular head and the centre of the lateral femoral condyle, and the lateral malleolus [35, 36]. The pins and clamps were adjusted until the limb was positioned parallel to the Plexiglass base with the stifle joint at an angle of 135°. The patella was attached to the cranially protruding femoral pin by creating a double loop of 3.5 metric polydioxanone (PDS) in the remaining insertion of the quadriceps muscle at the proximal patella and passing a rubber band^2^ between the suture loop and the pin four times. The tension created by the rubber loop was subjectively assessed as being sufficient to prevent any laxity in the patellar ligament. The length of the 4-fold rubber loop was between 34–43 mm in all specimens to minimize variability in the tension applied to the patellar ligament.

### Pre-osteotomy cage selection

A mediolateral pre-osteotomy radiograph was obtained to assess femoral and tibial position. Radiographs were obtained at 65kVp, 160mA and 100ms at a distance of 1m with a generator.^3^ A computed radiography cassette^4^ was processed in a computed radiography reader.^5^ The positioning was readjusted until the limbs were deemed to have minimal femoral or tibial rotation. The required cage size was determined using both the tibial plateau and the common tangent method [21]. If the two measurements indicated cage sizes that differed by two sizes based on manufacturer recommendations^6^, a cage of intermediate size was used (for example a 10.5mm cage was used if the common tangent method indicated a 9mm cage and tibial plateau slope indicated a 12mm cage). If the required cage sizes from each of the measurements were one size apart, with no intermediate size between the measurements, the larger cage was used.

### Osteotomy procedure

A tibial tuberosity osteotomy was performed perpendicular to the sagittal plane of the tibia from a point cranial to the medial meniscus to the distal aspect of the tibial tuberosity using an oscillating saw. Initially, the osteotomy was incomplete distally [11], leaving a cortical hinge of approximately 2-3mm thickness. The tibial tuberosity was advanced without breaking the distal cortical hinge and the pre-selected cage was placed into the osteotomy gap, with the distal edge of the cage placed at the level of the tibial tuberosity. A 0.9mm Kirschner wire was inserted into the tibial metaphysis through the caudal cage ear for stabilization of the cage within the osteotomy gap. A second mediolateral radiograph was obtained (0mm radiograph).

The osteotomy was then completed distally, and the tibial tuberosity was shifted proximally by 3mm. The tibial tuberosity was secured in place with large pointed bone reduction forceps placed perpendicular to the osteotomy plane with the distal end of the tibial tuberosity in contact with the tibial diaphysis. A third mediolateral radiograph was obtained. The same procedure was repeated with the tibial tuberosity translated proximally by 6mm.

### Radiographic assessment

Radiographs were checked to ensure that there had not been any movement or rotation of the specimen in relation to the external fixation and Plexiglass sheet, which could have changed the position of the specimen from the pre-osteotomy stage. Position of the cage was also assessed after each stage of proximalization. In most specimens the cage tended to shift slightly proximal during proximalization of the tibial tuberosity due to tilting of the caudally secured cage ear insert within the cage. This was deemed to be acceptable providing the cage had not tilted.

**Fig. 5:**
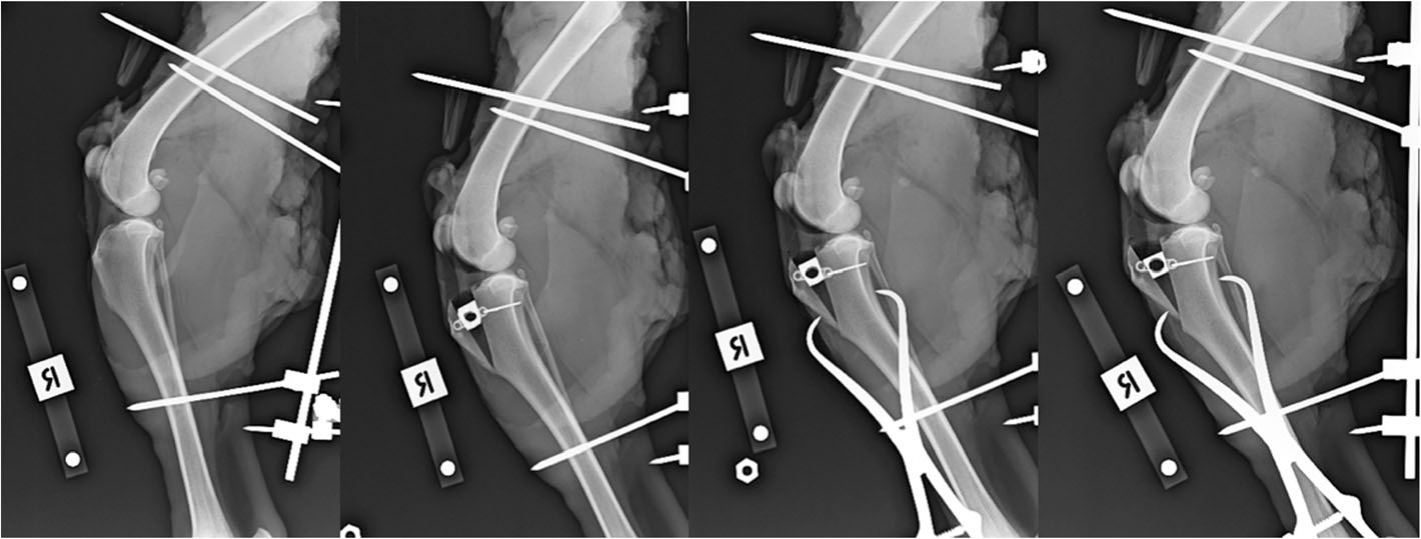
Series of the four lateral radiographs within one specimen prior to blinding. From *left* to *right*: Pre-osteotomy, and 0mm, 3mm, and 6mm proximalization of the tibial tuberosity

Once the series of pre-osteotomy, 0mm, 3mm and 6mm radiographs were obtained for each of the 6 specimens (Fig. 5), the radiographs were blinded. This was performed using a blinding method in which the 24 radiographs to be assessed were selected on eFilm^7^, exported to Osirix^8^, and cropped such that the distal aspect of the osteotomy including the absence or presence of pointed bone reduction forceps was no longer visible (Fig. 3). A random number generator^9^ was used to create a random sequence of all radiographs. Each radiograph was labelled with a number and all radiographs placed on a blank compact disc. This was presented to 3 observers; a specialist veterinary surgeon, a specialist veterinary radiologist, and a veterinary student for assessment of patellar position and patellar ligament angle.

A second set of all non-blinded images was randomised in the same way as described. The radiographs were calibrated and one observer (veterinary student) measured radiographic tibial tuberosity proximalization distance in the 3mm and 6mm stage radiographs with the measurement tool of the Osirix software^h^. The distance between the distal ends of the osteotomized edges of the tibial tuberosity and tibial metaphysis was measured on two separate occasions and averaged. This average was used to determine mean (+/-SD) tibial tuberosity proximalization distance for each stage.

### Patellar position measurement

**Fig. 6:**
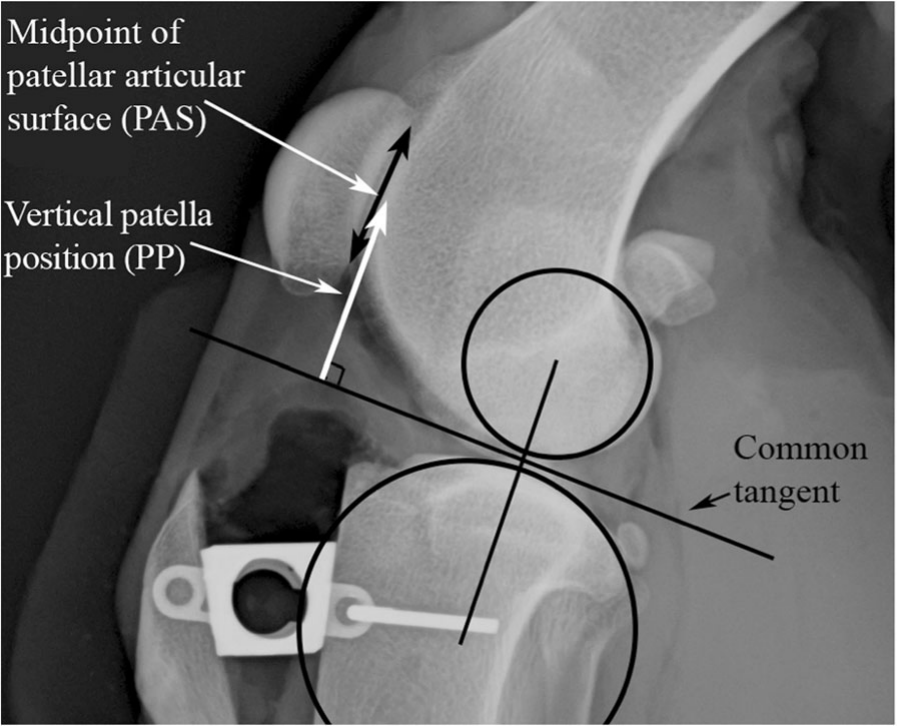
Vertical patellar position measurement. Patellar position was measured as the distance (mm) from the midpoint of the patellar articular surface perpendicular to the common tangent of femoral and tibial condyles. Note that this radiograph is blinded in that the degree of proximalization is not visible

Patellar position was measured using a modification of several indirect methods, mostly resembling the Blackburne-Peel method [26–28, 37]. Patellar articular length was measured from the base to the apex. A perpendicular measurement from the common tangent of the femoro-tibial articulation to the midpoint of the patellar articular surface was recorded (Fig. 6) and this measurement was used as the PP value.

### Patellar ligament angle measurement

Patellar ligament angle was measured using both tibial plateau slope (PLAslope) and the common tangent (PLAtangent) as a reference line to the patellar ligament. The tibial plateau slope was defined by the cranial and caudal extent of the medial tibial condyle [38]. The common tangent was evaluated by creating a line perpendicular to a line connecting the centres of superimposing circles corresponding to the respective articular surfaces of the femoral condyles and the medial tibial condyle.

### Statistical analysis

Statistical analysis was performed using a statistical software package.^10^ Data are presented as mean ± standard deviation (SD). Measurements from the three observers were pooled into pre-osteotomy, 0mm, 3mm and 6mm covariants for PP, PLAslope and PLAtangent. Shapiro-Wilk test was used to confirm normal distribution of the pooled data within each of the measurement stages (pre-osteotomy, 0mm, 3mm and 6mm) for PP, PLAtangent and PLAslope. The assumption of sphericity was confirmed for each of these stages using Mauchly’s Test of Sphericity. Repeated measures ANOVA was used to test for differences between pre-osteotomy PP and PP at the different proximalization stages, and to compare PLAslope and PLAtangent between the different proximalization stages. Pair-wise comparisons were used for PP, PLAslope and PLAtangent covariant measurement groups where results of the ANOVA were significant (P < 0.05) and post-hoc Bonferroni adjustments allowed for multiple comparisons within each of these categories.

Additionally, interobserver agreement was calculated based on intraclass correlation coefficient (ICC) analysis, measuring absolute agreement, where 0 represented no agreement/consistency and 1 represented perfect agreement/consistency. Excellent agreement was defined as ICC >0.8.

## Abbreviations

CrCL: Cranial cruciate ligament
MMP: Modified Maquet procedure
PLA: Patellar ligament angle
PLAslope: Patellar ligament angle derived from the tibial plateau slope
PLAtangent: Patellar ligament angle derived from the common tangent
PP: Patellar position
TTA: Tibial tuberosity advancement

## Footnotes

1 Small EK ESF Clamp, Imexx Veterinary Inc., Longview, Texas

2 Size 64 rubber band, Esselte Australia Pty Ltd, Sydney, Australia

3 Toshiba KXO-60G generator, Toshiba Asia-Pacific, Singapore, Singapore

4 35x43cm FCR Fuji IP, Type CC Cassette, Fujifilm, Tokyo, Japan

5 FCR Profect One Reader, Fujifilm, Tokyo, Japan

6 TTA preoperative planning guide, Kyon, Zurich, Switzerland

7 eFilm System 3.4, Merge Healthware, Chicago, Ilinois

8 32-bit DICOM viewer, Osirix, Geneva, Switzerland

9 Research Randomizer, Version 3.0, Retrieved on April 22, 2011, from http://www.randomizer.org/

10 SPSS20.0, IBM, New York, NY
